# Clinical and histological indicators for malignant transformation of oral submucous fibrosis: an analysis of cases from a tertiary care center

**DOI:** 10.1186/s12903-025-06443-y

**Published:** 2025-07-02

**Authors:** Vedaa Naik, Mathangi Kumar, Monica Charlotte Solomon, Chetana Chandrashekhar, Vasudeva Guddattu

**Affiliations:** 1 Manipal College of Dental Sciences, Manipal, Manipal Academy of Higher Education, 576104 Manipal, Karnataka India; 2 Department of Oral Medicine & Radiology, Manipal College of Dental Sciences, Manipal, Manipal Academy of Higher Education, 576104 Manipal, Karnataka India; 3 Department of Oral and Maxillofacial Pathology and Oral Microbiology, Manipal College of Dental Sciences, Manipal, Manipal Academy of Higher Education, 576104 Manipal, Karnataka India; 4 Department of Applied Statistics and Data Science of Public Health, Prassana School of Public Health, Manipal Academy of Higher Education, 576104 Manipal, Karnataka India

**Keywords:** Oral submucous fibrosis, Potentially malignant oral disorders, Malignant transformation, Histopathology

## Abstract

**Background:**

Oral submucous fibrosis (OSMF), a potentially malignant disorder predominantly affecting South Asian populations, is characterized by irreversible progression and significant malignant transformation potential. Early identification of risk indicators is crucial for implementing preventive measures and appropriate interventions to curb disease progression. While clinical parameters and histological features have been studied independently, comprehensive analyses evaluating multiple clinical manifestations, histopathological characteristics, and their correlations remain limited, particularly in long-term studies. This study aimed to assess individual clinical and histological parameters, and evaluate their relationships to identify early indicators of malignant transformation, thereby enabling more effective early intervention strategies.

**Methods:**

A retrospective analysis of electronic records from January 2012 to July 2024 was conducted on 118 OSMF patients aged 20–70 years, excluding those with concurrent mucosal lesions or incomplete records. Clinical parameters, including fibrous bands, burning sensation, mouth opening (measured by Vernier caliper), ulceration, and mucosal blanching, were graded based on Haider’s classification, while histopathological features such as epithelial thickness, keratinization, blood vessel characteristics, signet ring cells, inflammatory infiltration, and hyalinization were graded according to Pindborg and Sirsat’s classification. Data analysis was performed using Jamovi Software, employing descriptive statistics and chi-square tests to assess categorical data, with statistical significance set at *p* < 0.05.

**Results:**

Among 118 OSMF patients (89.8% males, mean age 41 years), buccal mucosa was predominantly affected (93%). Major clinical features included burning sensation (87.3%), blanching (95.8%), and fibrous bands (96.6%). Of 106 patients, 41.5% showed Stage 1 mouth opening (> 20 mm), with 60% of malignant transformations occurring in this group. Histopathologically, 49.2% cases were moderately advanced, showing atrophic epithelium (56%), keratinization (85.6%), and juxta-epithelial hyalinization (92.4%). The study revealed a 4.2% malignant transformation rate, with epithelial dysplasia observed in 27% cases.

**Conclusions:**

The study highlights the significance of early diagnosis, as even in the initial stages of OSMF (mouth opening < 20 mm, grade 1), there were advanced histological changes that were observed along with a risk of malignant transformation. The observed rate of malignant transformation was 4.2% and hence prompt identification of the clinical and histological indicators for malignant transformation can help in improving patient outcomes.

## Background

Oral Submucous fibrosis (OSMF) is a potentially malignant oral disorder that occurs due to habitual consumption of areca nut [1, 2]. This chronic condition causes a disruption in the wound healing process following prolonged persistent injury to the oral mucosa consequently affecting parts of the oral cavity, esophagus and pharynx [3, 4]. The well-established contributing factors for the malignant transformation of OSMF are genetic predisposition, vitamin deficiency, and excessive chilli consumption. The International Agency for Cancer Research (IACR) has classified the areca nut as class 1 carcinogen which contains cytotoxic and mutagenic components. This leads to dysplastic changes, mucosal stiffness, and functional morbidity of oral tissues. Furthermore, these agents heighten stressors and angiogenic factors like TNF-alpha, IL-1, and reactive oxygen species, thereby increasing susceptibility to malignant transformation [2, 3, 5].

There has been an observed increase in the incidence of OSMF, coupled with a discernible shift in the median age of onset toward younger individuals. This phenomenon can be attributed to the widespread availability and prevalent use of various commercial products containing areca nut and tobacco [6, 7, 8]. OSMF represents a significant global health concern, with an estimated prevalence of 4.47% worldwide and a notably higher rate of 6.63% in India [7]. Over the past four decades, India has witnessed a dramatic surge in OSMF cases, with prevalence escalating from 0.03 to 6.42% [3]. The condition’s progressive nature and its potential for malignant transformation make it a critical focus in oral healthcare, particularly in the Indian subcontinent. Systematic reviews have revealed higher malignant transformation rates (MTR) in this region compared to areas like China and Taiwan, with an overall transformation rate of 6% [5]. These regional disparities are primarily attributed to variations in areca nut preparations, consumption patterns, and tobacco use, highlighting the crucial role of geographical and habit-specific risk factors in disease progression [5].

The transformation from OSMF to oral cancer can vary depending on factors such as age, gender, usage patterns, and diagnostic criteria. Despite these differences, most studies have reported that a subset of OSMF cases progresses to oral cancer [8]. OSMF-derived malignant tumors tend to exhibit greater clinical invasiveness, a higher risk of metastasis, and an increased likelihood of recurrence [3].

A 10-year retrospective analysis revealed that oral squamous cell carcinoma arising from OSMF typically affects younger patients and presents distinct clinicopathological features compared to conventional OSCC [9]. Furthermore, a 9-year retrospective study demonstrated that mucosal changes peak during moderate stages of the disease, while burning sensation shows progressive worsening, emphasizing the critical importance of early detection through comprehensive clinical examination for preventing disease progression and reducing malignant transformation risk [10]. Yet another comparative study by Shivakumar et al. involving 50 OSMF patients used the Khanna and Andrade classification to evaluate the relationship between clinical and histopathological features. They found a significant correlation between clinical severity and microscopic changes, while noting that mild clinical cases could show severe histological alterations. The study highlighted the importance of thorough clinical and histopathological evaluation for accurate diagnosis, treatment planning, and early intervention [11]. Several studies, including those by Motgi AA. et al., Biradar BS et al., Pandiar D et al., and Abidullah M et al. have demonstrated the importance of correlating histopathological and clinical staging in OSMF [12, 13, 14, 15].

While many studies have offered valuable insights into the clinical and functional aspects of OSMF, fewer have incorporated detailed histopathological evaluation, which is crucial for gaining a more comprehensive understanding of the disease’s progression. Owing to the irreversible nature of the lesion, it is crucial to prioritize prevention through early diagnosis to control the disease and hinder its progression into malignancy [3].

The present study aims to evaluate both clinical and histopathological features comprehensively, aiming to identify the clinical and histological predictors for malignant transformation in patients with OSMF. By evaluating multiple parameters together, we aim to understand the trends that can contribute to earlier diagnosis and more informed intervention.

## Materials and methods

This study involved the analysis of digital electronic medical and dental records of subjects diagnosed with oral submucous fibrosis. Kasturba Medical College and Kasturba Hospital Institutional Ethics Committee approved the conduct of this study (IEC2-378/2022). The need for written informed consent was waived because of the retrospective nature of this study. Patient records between January 2012 to July 2024 diagnosed with OSMF were analyzed. Participants aged 20 to 70 years of both genders, with clinical and histopathological features consistent with OSMF, were included in the study. Subjects with other mucosal lesions along with OSMF were excluded from the study. Also, patient case sheets with incomplete details were excluded from the study. The following parameters were systematically documented for each patient; demographic data including age and gender, oral abusive habit history (type and duration) site of occurrence of lesion. The data on the presence/ absence of the clinical features like burning sensation, fibrous bands (location), mouth opening, ulcers, marbled appearance, shrunken uvula, depapillation of tongue and restricted tongue mobility were retrieved from that patient records. It is well-established that these clinical diagnostic parameters are employed for the clinical staging of OSMF. The study subjects were categorized based on age as follows:

- Group 1: <20 years.
- Group 2: 21–50 years.
- Group 3: >50 years.

The clinical examination and documentation that were performed by an experienced Oral Medicine specialist was retrieved from the patient records. The histopathological diagnosis was performed by an experienced pathologist. The clinical and functional staging of OSMF was documented according to the classification proposed by Haider et al. [16]. The clinical stage is based on the location of the bands (1. Faucial bands only, 2. Faucial and buccal and 3. Faucial, buccal and labial bands) while the functional staging is based on the mouth opening (A: ≥ 20 mm; B: 11–19 mm; C: ≤ 10 mm) [16]. The histopathological grading was based on the classification by Pindborg and Sirsat [17]. This system typically accounts the changes in the epithelium (atrophic oral epithelium secondary to the connective tissue changes; intercellular edema; epithelial atypia; signet cells, loss of rete pegs) and the oral connective tissue (inflammatory cell infiltration, narrowing of blood vessel; juxta epithelial hyalinization) in OSMF [17].

The standard clinical protocol that is employed for patients with OSMF in the Oral Medicine Department of our tertiary care center involves careful systematic documentation of the clinical symptoms, clinical examination findings (mouth opening, presence and location of the fibrous bands, presence/ absence of ulceration and mucosal blanching). This is followed by clinical staging of OSMF based on the clinical criteria for grading. The mouth opening is measured using the Vernier caliper and this corresponds to the inter-incisal distance (measured in millimeters). This is the distance between the mesio-incisal angles of the maxillary and mandibular central incisors. This value is documented systematically in the case sheet of every patient with OSMF which was utilized for this study purpose.

Histopathological analysis of all the reports of included patients involved the assessment of the following criteria:

- Epithelial thickness, keratinization, blood vessel constriction, signet ring appearance, inflammatory cell infiltration, epithelial hyalinization.

The clinical and histopathological parameters of OSMF were systematically documented for each patient and entered in a Microsoft Excel spreadsheet.

### Statistical analysis

The data were entered in a Microsoft Excel spreadsheet, and statistical analyses were conducted using Jamovi Software for Windows (Version 2.6.13) [Sydney, Australia]. Descriptive statistics were employed to present the data, utilizing frequency and percentage analysis, while continuous variables were described using means and standard deviations. Chi-square tests were applied to assess the significance of qualitative categorical data. A probability value of 0.05 was considered significant in the statistical tools used.

## Results

### Demographic characteristics of study subjects

This cross-sectional study consisted of a total of 118 patients with oral submucous fibrosis who were included for data analysis. There were 106 (89.8%) males and 12 (10.2%) were females. The average age of the participants in this study was 41 years. The patients were categorized into five age groups: 0(0%) were under 20 years (Group 1); 92 (77.9%) were aged 21–50 years (Group 2); and 26 (22%) were > 50 years old (Group 3).

### Site of lesions observed in the study subjects

The lesions were most frequently observed at the following sites, listed in descending order: buccal mucosa, affecting 110 patients (93%); palate, seen in 38 patients (32%); labial mucosa, involved in 37 patients (31%); tongue, noted in 28 patients (24%); faucial pillars, observed in 21 patients (18%); floor of mouth, present in 16 patients (14%); and gingiva, the least affected site, found in 8 patients (7%). Many individuals exhibited lesions at more than one site. (Table 1)

**Table 1:** Demographic details of the subjects

Age	
*Age range*	*N(%)*
< 20 years	0 (0)
21–30	92 (77.9)
> 50	26 (22.0)
**Gender**	
Male	106 (89.8)
Female	12 (10.2)
**Severity of OSMF based on the site of involvement**	
Buccal Mucosa	110 (93.2)
Palate	38 (32.2)
Labial Mucosa	37 (31.4)
Tongue	28 (23.7)
Faucial pillars	21 (17.8)
Floor of the Mouth	16 (13.6)
Gingiva	8 (6.8)
**Habit Duration**	
Mean (SD); Range	9.5 (7.3); 1.0–30.0 years

### Clinical staging of the study subjects based on the mouth opening

Mouth opening was clinically examined and recorded for 106 participants, with detailed information documented in their clinical files, though data for 12 patients was unavailable due to missing entries in the records. A total of 44 (41.5%) patients were recorded with an interincisal mouth opening > 20 mm. This was which were diagnosed as Stage 1 (Haider et al.). A total of 38 (35.8%) patients were categorized into Stage 2 OSMF and the remaining 24 (22.6%) patients recorded an interincisal opening of < 10 mm and were classified as Stage 3 OSMF.

### Clinical characteristics of study subjects

History revealed that 103 (87.3%) of the total patients exhibited burning sensation. Blanching was observed in 113 (95.8%) of the patients and fibrous bands were present in 114 (96.6%) patients (Table 2). In 73 of the 118 patients, data were available for the uvula, tongue depapillation, and tongue mobility, with a shrunken uvula observed in 42 patients (57.5%), depapillation in 28 (38.4%), and restricted tongue mobility in 34 (46.6%), while similar data for the remaining patients were not recorded.

**Table 2:** Distribution of patients of OSMF according to various clinical features

Clinical Features	*N*(%)
*Burning Sensation*	
Present	103 (87.3)
Absent	15 (12.7)
*Fibrous Bands*	
Present	114 (96.6)
Absent	4 (3.4)
*Mouth Opening*	
Restricted	103 (87.3)
Non- restricted	15 (12.7)
**Mouth Opening (staging according to Haider at al.)** *Total Patients = 105 Missing data = 13*	
Grade I (> 20 mm)	44 (41.9)
Grade II (11–19 mm)	37 (35.2)
Grade III (< 10 mm)	24 (22.9)

### Histopathological characteristics of the study subjects

The histopathological grading system for OSMF which was documented in the final histopathological reports (signed by senior pathologists: Author MC and CC) were retrieved from the records of the included patients. The grading system that was mentioned in the final reports was noted and were systematically documented for each patient.

Patients were categorized into four stages based on histologic features, following the grading system proposed by Pindborg and Sirsat (1966) [18]. This classification included 6 patients (approximately 5.1%) in the very early stage; 38 patients (32.2%) in the early stage; 58 patients (49.2%) in the moderately advanced stage; and 11 patients (9.3%) in the advanced stage. Additionally, 5 patients were found to have undergone malignant transformation of their OSMF lesions, resulting in a malignant transformation rate of 4.2% in this study.

**Fig. 1:**
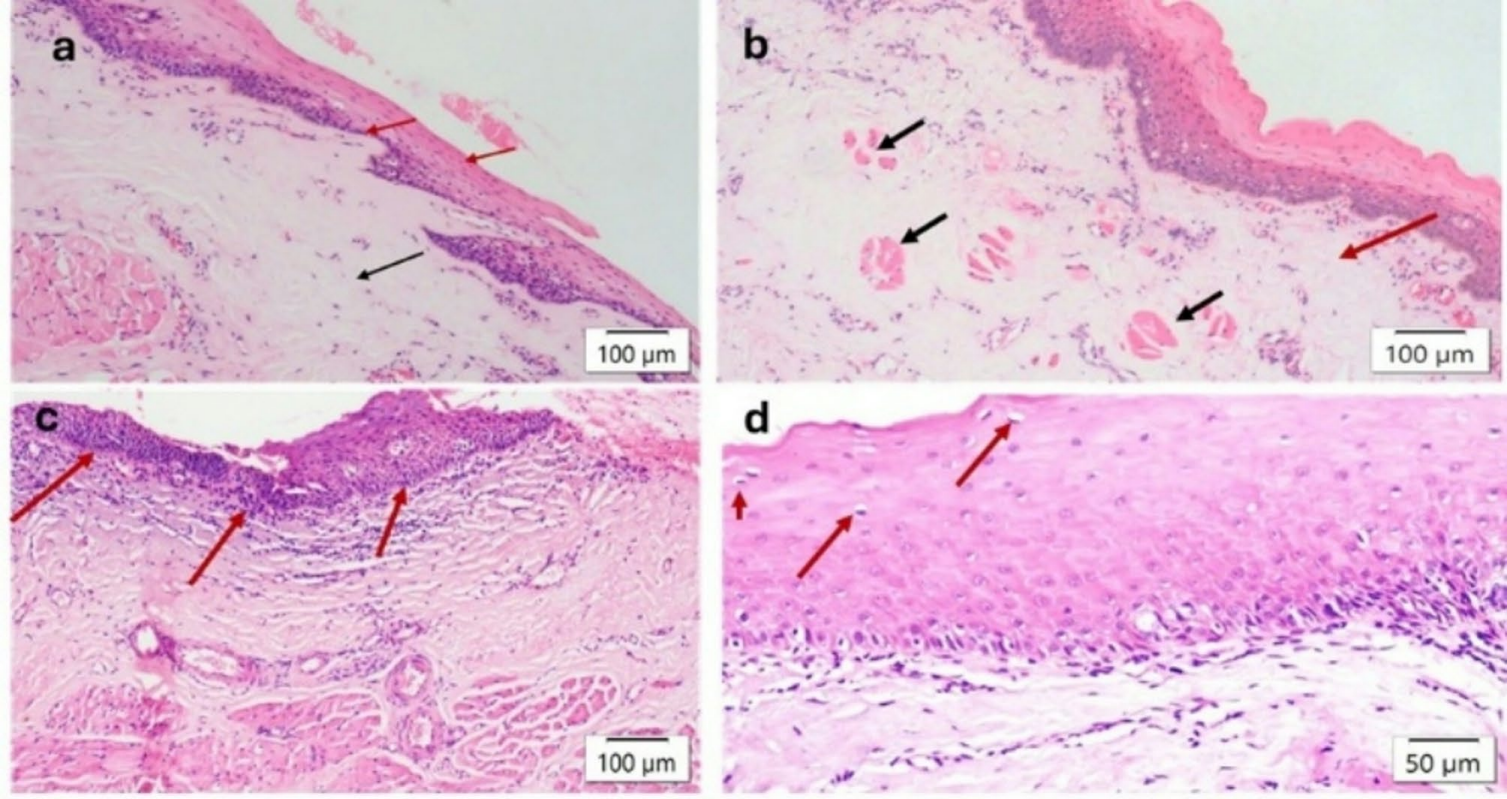
(**a**) Photomicrograph showing an atrophic epithelium with loss of rete ridges (red arrows) with juxta epithelial hyalinization (black arrow) (H&E 10X). (**b**) Photomicrograph showing a juxta epithelial hyalinization (red arrow) and degeneration of muscle tissue (black arrows) (H&E 10X). (**c**) Photomicrograph showing dense inflammatory infiltrate of lymphocytes (red arrows) (H&E 10X). (**d**) Photomicrograph showing signet cells in the epithelium (red arrows) (H&E 20X) in oral submucous fibrosis

**Fig. 2:**
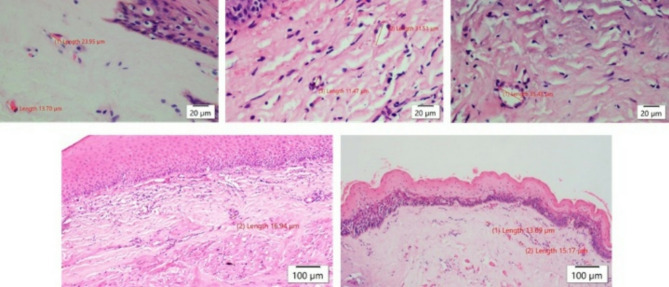
Photomicrograph showing variations in the diameters of the vascular channels in the study subjects with oral submucous fibrosis (H&E 100 μm: 10X, 20 μm: 40X)

Histopathological sections were categorized into three groups based on epithelial thickness: acanthotic, present in 45 patients (38%); atrophic, seen in 66 patients (56%) (Fig. 1.a); and both atrophic and acanthotic areas, identified in 7 patients (6%). Among all participants, 101 (85.6%) exhibited keratinization in their histological sections. The signet ring appearance was found in only 12 (10.2%) sections. Juxta-epithelial hyalinization was detected in the connective tissue of 109 (92.4%) participants (Fig. 1.a) (Fig. 1.b). Chronic inflammatory infiltrate, predominantly composed of lymphocytes, was dense in 96 (81.4%) histological sections (Fig. 1.c). Indications of moderate vascularity, including constricted blood vessels, were noted in 23 (19.5%) sections (Fig. 2). Mild to moderate features of dysplasia were observed in 27% of the study population, including nuclear and cellular pleomorphism, hyperchromatic nuclei, altered nuclear-cytoplasmic ratio, and multiple prominent nucleoli (Table 3). The correlation between mouth opening grade (Haider et al.) and histological grade is depicted in Table 4. The correlation between age and histological grade of the study subjects is depicted in Table 5. The correlation between vascular channels and histological grade is depicted in Table 6. The correlation between malignant transformation and inflammatory infiltrate is depicted in Table 7. The relationship between malignant transformation, hyalinization and signet ring appearance of in the histological sections of the study subjects is depicted in Table 8. The correlation between malignant transformation and depapillation of the tongue is depicted in Table 9.

**Table 3:** Distribution of patients of OSMF according to various histological features

Histological Features	*N*(%)
*Hyalinization*	
Present	109 (92.4)
*Vascular Channels*	
Constricted	23 (19.5)
*Keratinization*	
Present	101 (85.6)
*Inflammatory infiltrate*	
Dense	96 (81.4)
Sparse	22 (18.6)
*Signet Ring Appearance*	
Present	12 (10.2)
*Dysplastic Features*	
Present	32 (27.1)
*Epithelial Thickness*	
Atrophic	66 (55.9)
Acanthotic	45 (28.1)
Both atrophic and acanthotic	7 (5.9)
*Distribution of Subjects according to the Histological grade (Pindborg and Sirsat*,* 1966)*	
Very early	6 (5.1)
Early	38 (32.2)
Moderately advanced	58 (49.2)
Advanced	11 (9.3)
Malignant transformation	5 (4.2)

**Table 4:** Correlation between mouth opening grade (Haider et al.) and histological grade

Histological grade	Very early	Early	Moderately advanced	Advanced	Malignant transformation	Total	*P* value
**Mouth Opening Grade I**	2 (66.7)	21 (61.8)	15 (27.8)	3 (33.3)	3 (60.0)	44 (41.9)	0.106
**Mouth Opening Grade II**	1 (33.3)	6 (17.6)	25 (46.3)	4 (44.4)	1 (20.0)	37 (35.2)	
**Mouth Opening Grade III**	0 (0.0)	7 (20.6)	14 (25.9)	2 (22.2)	1 (20.0)	24 (22.9)	

**Table 5:** Correlation between age and histological grade of patients

Histological grade	Very early	Early	Moderately advanced	Advanced	Malignant transformation	Total	*p* value
**Total N(%)**	6 (5.1)	38 (32.2)	58 (49.2)	11 (9.3)	5 (4.2)	118	0.067
**Age (Mean)**	45.5	42.3	39.2	47.3	49.6	41.7	

**Table 6:** Correlation between vascular channels and histological grade of patients

Histological grade	Very early	Early	Moderately advanced	Advanced	Malignant transformation	Total	*p* value
**Presence of constricted blood vessels**	2 (8.7)	3(13.0)	7(30.4)	8(34.8)	3(13.0)	23(19.5)	< 0.001
**Absence of constricted blood vessels**	4(4.2)	35(36.8)	51(53.7)	3(3.2)	2(2.1)	95(80.5)	

**Table 7:** Correlation between malignant transformation and inflammatory infiltrate

Malignant Transformation (MT)	MT Present	MT Absent	Total	*P* value
**Total N (%)**	113 (95.8)	5 (4.2)	118	0.612
**Patients in whom Inflammatory infiltrate present (%)**	5(100)	91(80.5)	96(81.4)	
**Patients in whom Inflammatory infiltrate absent (%)**	0(0.0)	22(19.5)	22(18.6)	

**Table 8:** Correlation of mouth opening with malignant transformation, hyalinization and signet ring appearance

	*Malignant Transformation* *Total subjects = 118*			*Hyalinization* *Total subjects = 118*			*Signet Ring Appearance* *Total subjects = 118*			*Epithelial Thickness* *Total subjects = 106; Missing data = 12*			
	*Present*	*Absent*	*p value*	*Present*	*Absent*	*p value*	*Present*	*Absent*	*p value*	*Atrophic*	*Acanthotic*	*Both*	*P value*
***No. of subjects (%)***	5 (4.7)	101 (95.3)	0.173	97 (91.5)	9 (8.5)	0.053	12 (11.3)	94 (88.7)	0.944	58 (54.7)	41 (38.7)	7(6.6)	0.596
***Mean Mouth Opening in millimeters (SD)***	23.6 (10.6)	18.0 (8.8)		17.7 (8.8)	23.8 (8.7)		18.1 (8.6)	18.3 (9.0)		19.1 (10.1)	17.4 (7.3)	16.7 (8.5)	

**Table 9:** Correlation between malignant transformation and depapillation of tongue

Malignant Transformation	Present	Absent	Total	*P* value	Missing data
**Total N (%)**	3 (4.1)	70 (95.9)	73	0.430	45
**No. of subjects with presence of depapillation of tongue (%)**	0 (0.0)	28 (40.0)	28 (38.4)		
**No. of subjects with absence of tongue depapillation (%)**	3 (100.0)	42 (60.0)	45 (61.6)		

## Discussion

OSMF represents one of the most frequently encountered oral potentially malignant disorders (OPMD) with increased prevalence subsequent to its identification as having malignant potential by Paymaster in 1956 [1, 19]. It exhibits notable prevalence among South Asian and Southeast Asian populations and has extended its incidence to diverse geographic regions worldwide, including the United Kingdom, the United States, South Africa, and numerous other countries [20, 21, 22]. Many individuals diagnosed with OSMF often endure a diminished quality of life, alongside functional impairments like masticatory dysfunction and impaired speech, which subsequently contribute to psychological challenges and distress in social contexts [10, 23].

The elevated concern is also due to the fact that it is regarded as having a greater propensity for progression to oral squamous cell carcinoma (OSCC) as compared to other documented OPMDs [24, 25]. In a study by Merchant et al. a 19.1 fold higher likelihood of developing oral squamous cell carcinoma in comparison to those without the condition was revealed [26]. As OSMF transitions into OSCC, the 5 year survival rate for OSCC diminishes to less than 60%. Hence, early diagnosis and prevention are imperative to mitigate and effectively address the malignant progression of OSMF [27]. The malignant transformation of OSMF correlates with a variety of factors, including various clinical and histological features.

Long-term prospective studies with large cohorts and follow-up assessments on the role of these features in malignant transformation are relatively limited in literature. This research aims to provide a more thorough understanding of the clinical and histopathological predictors of malignancy in OSMF. In doing so, we intend to contribute valuable data in identifying the clinical and histological indicators for malignant transformation of OSMF. The study involved a comprehensive analysis of case records from 118 subjects who were all clinically and histologically diagnosed with OSMF.

The current study has reported a higher prevalence among males, with a male is to female ratio of 9:1 which is consistent with findings observed in studies conducted by Kizhakkoottu et al., Chaudry et al., Yang et al., Chourasia et al., and Sumathi et al. This could be due to more frequent habitual consumption of areca nut among the male population [28, 29, 30, 31, 32]. This differs from the research conducted by Pindborg et al. and Rajendran et al., which indicate a female predisposition [33]. The mean (SD) age of participants in this study was 41.7(11.4) years, with the youngest being 22 and the oldest 68 years of age. Majority of participants fell within the 31–40 age group, accounting for 29.9% of the total.

OSMF clinically progresses from erythema and a marble-like blanched appearance to the formation of fibrous bands in advanced stages. Fibrosis being the key element of OSMF results from increased collagen accumulation due to heightened production and reduced breakdown, influenced by genetic polymorphisms and autoimmune factors. Areca nut alkaloids modify fibroblast phenotypes, leading to increased collagen secretion and cross-linking via lysyl oxidase. Chronic irritation from areca nut chewing activates T cells and macrophages, promoting cytokine and growth factor release that enhances collagen production and inhibits degradation, thus worsening fibrosis [34]. In this study, blanching was observed in 95.8% of the study participants, whereas fibrous bands were identified in 96.6% of the cohort.

An early alteration in the mucosa is the appearance of vesicles, which gradually break down into erosions, eventually leading to ulceration [10]. The present study reported ulceration in 25.5% of the study population out of which approximately 32% belonged to stage I OSMF (Haider et al.) The presence of ulceration in the early stages of OSMF suggests that mucosal damage may begin at the onset of the disease, even before it progresses to more severe stages. In line with the classifications by Gupta et al. (1980) and Khanna and Andrade (1995), ulceration is identified in the early stages of disease progression. This further supports the idea that mucosal damage, such as ulceration, begins early in OSMF, reinforcing its presence as an initial marker of tissue stress before the condition worsens [35, 36].

Sites of occurrence of lesion included the buccal mucosa, tongue, labial mucosa, faucial pillars, floor of the mouth, palate, and gingiva. The buccal mucosa exhibited the highest frequency of involvement, whereas the gingiva was the least affected. It was also common for patients to present with lesions at multiple sites. Fibrosis may advance posteriorly to encompass the soft palate and uvula, resulting in a distinctive hockey stick or a shrunken uvula appearance [33]. In the present analysis, data regarding the assessment of a shrunken uvula was available for 73 subjects, of whom 42 (57.7%) exhibited this feature.

A common symptomatic feature in patients with OSMF is a burning pain or sensation in the oral mucosa, often one of the earliest complaints. This discomfort is typically exacerbated by the consumption of spicy foods. In the current study, a total of 87.3% of subjects reported to have burning sensation with a mean VAS score of 5. The pathogenesis of burning pain in OSMF in the recent literature has been seen to be multifactorial. One mechanism involves chemicals in areca nut activating mast cells, which sensitizes nerve endings to capsaicin. Another pathway suggests direct injury to nerve endings by these chemicals, causing burning pain [37]. Increased epithelial atrophy also reduces the distance of intra-epithelial nerve endings from the surface, further contributing to this sensation [38, 39, 40]. Capsaicin’s role in OSMF is supported by evidence showing its activation of TRPV-1 receptors on C fibers, leading to continuous burning pain [37]. Nagesh and Bailoor (1993) classified burning sensations in OSMF stages: in stage 1, it occurs with spicy foods or hot beverages; in stage 2, it appears without stimuli; and in stage 3, it becomes more severe, affecting daily activities [41].

The progressive decline in mouth opening, a pivotal clinical parameter, is attributed to submucosal and muscle fibrosis, serving as an indicator of disease severity [42, 43]. Within this study, 87.3% of the study population had restricted mouth opening. The average mouth opening as seen in our study was 18 mm. Approximately 60% of patients had a mouth opening of < 20 mm in the present study. It has been suggested that individuals with mouth.

The analysis of 105 subjects whose mouth opening grades were analyzed in the study. Grade 1 restriction was predominant (44 cases, 41.9%) across all stages, with a notable finding that 3 out of 5 cases (60%) with malignant transformation presented with only Grade 1 restriction (> 20 mm). The presence of malignant transformation in cases with minimal mouth opening restriction emphasizes that mild restriction does not preclude advanced disease. The Grade 2 mouth opening (11–19 mm) showed highest prevalence in moderately advanced and advanced histological stages (25/54 cases, 46.3% and 4/9 cases, 44.4% respectively), while Grade 3 mouth opening maintained a relatively consistent distribution across stages 1 through 5 (ranging from 20 to 25.9%). Furthermore, the predominance of cases in the moderately advanced stage (58 cases, 49.2%) with variable grades of mouth opening restriction suggests potential delays in initial presentation. The lack of statistical significance (*p* = 0.106) reinforces that mouth opening restriction alone may not be a reliable indicator for disease progression, emphasizing the need for comprehensive clinical examination and regular screening regardless of mouth opening status.

No significant correlation was observed between patient age and histological staging of the disease, suggesting that younger individuals may present with advanced histological stages, while older individuals may exhibit less advanced stages, or vice versa. A similar conclusion was drawn in a study by Pandiar et al. (2023), which classified 238 cases of OSMF using Pindborg and Sirsat’s 1966 classification and found no age-based correlation across any of the histological stages of OSMF [13].

In the present study, hyperkeratosis was observed in 85.6% of cases, manifesting as parakeratosis, orthokeratosis, or a combination of both patterns. Oral mucosa displays diverse responses to different kinds of stimuli. Recent literature has suggested that both chemical and mechanical stimuli, such as the consumption of areca nut and slaked lime, prompt keratinocyte differentiation, possibly resulting in hyperkeratosis in OSMF due to disturbances in the cornification process of the oral epithelial barrier [13, 44]. It has been observed that loricrin, an advanced differentiation indicator in fully matured keratinocytes, is more prominently expressed in keratotic oral epithelium and OSMF than in normal epithelial tissue, where it is usually absent. This presence could suggest underlying protective mechanisms linked to chewing practices [44].

Signet ring cell appearance is characterized by large cytoplasmic vacuoles compressing the nucleus into a crescent shape [45]. This microscopic feature, commonly found in OSMF, has been well-documented in literature. Signet cells were observed in 10.2% of the cases in the present study (Fig. 1.d). The findings of this study closely parallel those of classical studies by Pindborg and Sirsat, who reported a prevalence of 13%, and Pindborg’s subsequent analysis of 53 biopsy samples (1970) showing 19.3% occurrence throughout the epithelium [18, 46]. The mean mouth opening (18.1 mm) did not vary significantly with the presence of signet cells on histopathological examination in the study subjects. However, contemporary research by Pandiar et al. (2023), examining a larger sample size of 238 cases, reported a substantially higher prevalence of 41.6%. Recent literature suggests these cellular alterations may result from mechanical stress such as friction provoked epithelial changes [13].

In OSMF, epithelial modifications occur, starting with hyperplasia in the early phases and progressing to atrophy in the later stages, potentially due to microtrauma caused by areca nut chewing, which compromises the epithelial barrier. These changes may also be linked to pathological modifications in the lamina propria, such as increasing hyalinization, decreased vascular supply, and reduced cellularity, ultimately leading to ischemia [47, 48]. The present analysis revealed that atrophic epithelium was identified in 55.9% of cases, acanthotic epithelium in 38.1%, while a combination of both types was observed in 5.9% of cases.

It has been hypothesized that diminished vascularity, resulting in tissue hypoxia, leads to the accumulation of carcinogens on the epithelial surface. Due to impaired penetration and lack of systemic absorption, these carcinogens persist locally and interact with the atrophic epithelium. This prolonged exposure is thought to induce dysplastic changes within the epithelium, contributing to the progression toward malignancy [13, 49]. Research by Pindborg and others has shown that epithelial dysplasia in OSMF cases occur at a rate between 7% and 26% [46, 50, 51, 52]. In our study, the prevalence was 27.1%, consistent with this range. The blood vessels appeared constricted in subjects with later stages of OSMF than early lesions in this study. However, some studies, such as that by Jayasooria et al. (2011), have reported rates as high as 43% [49].

In our study, juxta-epithelial hyalinization was observed in 92.4% of the participants, a phenomenon attributed to cross-linking of collagen caused by disruptions in collagen metabolism and extracellular matrix remodeling [53]. As collagen deposition and cross-linking progress, fine collagen bundles in the initial stages gradually transform into complete hyalinization, where no distinct bundles are visible in the advanced stages. This progression highlights its role as a key pathological change, making it an early and reliable histopathological marker for identifying OSMF [54].

A diffuse chronic inflammatory infiltrate, a hallmark of OSMF consisting of lymphocytes, monocytes, and occasionally neutrophils and eosinophils, was observed in 81.4% of cases, while the remaining 18.6% showed only sparse infiltration. In a study by Isaac U et al. (2008), where 35 biopsy samples were analyzed, the inflammatory infiltrate was present in 100% of the cases, though this could be due to the small sample size [55].

The literature indicates that vascularity varies across different stages of OSMF, with early stages typically showing increased vascularity due to chronic inflammation and hypoxia-induced angiogenesis. In contrast, advanced stages are often associated with reduced vascularity as fibrosis progresses, restricting angiogenic activity [53]. This study observed a range of vascular patterns, where 19.5% of cases had constricted blood vessels, while 80.5% exhibited dilation. Notably, 34.8% of advanced cases still showed constricted vessels, which contrasts with some reports that describe dilated vessels in later stages [56, 57]. This suggests a more complex and dynamic vascular response during disease progression. The strong statistical significance (*p* < 0.001) shown in our study in the correlation between the constriction of vascular channels and the histological grade suggests that the presence of constricted blood vessels is strongly associated with histological grade and could potentially be an important marker for disease progression or severity.

Vasodilation in advanced stages of OSMF may be linked to malignant transformation, as suggested by literature. Dense fibrosis limits nutrient and oxygen supply, prompting dilation of blood vessels to meet the increased demands of transforming cells [58]. This process is driven by hypoxia and HIF-1α, which stimulate angiogenesis. In our study, 2% of advanced OSMF cases exhibited vasodilation, and notably, 2.1% of cases that underwent malignant transformation also had dilated vessels. This supports the idea that vascular changes, though limited in occurrence, could be indicative of malignant progression in these patients [53, 58, 59].

Since its recognition as a condition having malignant potential nearly seven decades ago, OSMF has remained a significant concern in oral oncology. Our current investigation, revealing a malignant transformation rate of 4.2%, contributes meaningful data to this ongoing discussion. This finding aligns with the spectrum of transformation rates documented across various populations. Notably, a landmark 17-year follow-up study by Murti and colleagues reported a transformation rate of 7.6%, while regional Indian studies from Kolkata and Nagpur documented rates of 2.6% and 3.3% respectively [60, 61].

Chinese epidemiological data suggests relatively lower rates, hovering between 1 and 4%, with a slight male predisposition [62, 63, 64]. The Indian subcontinent presents a different scenario, with studies from various regions reporting transformation rates between 2.5% and 8%. Taiwanese research has documented transformation rates of 5.4% for OSMF with epithelial dysplasia (mean duration 40 months) and 1.9% for OSMF alone. A subsequent Taiwanese study found similar results with 4.84% transformation in OSMF with dysplasia and 3.72% in OSMF alone. Our observed transformation rate of 4.2% falls well within the established range, as outlined above, further contributing to the growing body of evidence on OSMF’s malignant potential.

The present study explored clinical and histological predictors for malignant transformation and this data is valuable for improved patient outcomes. The limitation of the present study is the cross-sectional retrospective nature of the study due to which there was lack of complete data in certain patient records. This could create small pitfalls for which the clinical and histological predictors cannot be completely assessed. Also, due to the absence of follow up data, it is hard to estimate the true progression of the disease from a cross-sectional analysis of this study. Since the statistical significance is inconsistent across findings owing to the study design, causal inferences cannot be conclusively established. Further prospective longitudinal studies in this cohort of patients are essential to conclusively establish causal inferences of these findings.

## Conclusion

The study underscores the critical importance of early diagnosis and vigilant monitoring, as even clinically mild cases (mouth opening < 20 mm- grade 1) were seen to demonstrate advanced histological features and malignant transformation. The observed rate of malignant transformation was 4.2% and hence prompt identification of the clinical and histological indicators for malignant transformation can help in improving patient outcomes. These indicators are crucial for timely intervention and management, potentially preventing progression to more severe stages or malignancy.

## Data Availability

The dataset supporting the conclusions of this article is included within the article. However, additional information can be requested from the corresponding author upon reasonable inquiry.

## Abbreviations

OSMF: Oral submucous fibrosis
